# Tuning Corrosion Resistance and AC Soft Magnetic Properties of Fe-Co-Ni-Al Medium-Entropy Alloy via Ni Content

**DOI:** 10.3390/e26121038

**Published:** 2024-11-30

**Authors:** Wenfeng Peng, Yubing Xia, Hui Xu, Xiaohua Tan

**Affiliations:** Institute of Materials, School of Materials Science and Engineering, Shanghai University, Shanghai 200072, China; wen22722361@163.com (W.P.); xiayubing1004@163.com (Y.X.)

**Keywords:** medium-entropy alloys (MEAs), soft magnetic properties, corrosion resistance, phase constitution, grain boundary distribution

## Abstract

Corrosion of soft magnetic materials during service can significantly impact their performance and service life, therefore it is important to improve their corrosion resistance. In this paper, the corrosion resistance, alternating current soft magnetic properties (AC SMPs) and microstructure of FeCoNi*_x_*Al (*x* = 1.0–2.0) medium-entropy alloys (MEAs) were studied. Corrosion resistance is greatly improved with an increase in Ni content. The *x* = 2.0 alloy has the lowest corrosion current density (*I_corr_* = 2.67 × 10^−7^ A/cm^2^), which is reduced by 71% compared to the *x* = 1.0 alloy. Increasing the Ni content can improve the AC SMPs of the alloy. When *x* = 1.75, the total loss (*P_s_*) is improved by 6% compared to the *x* = 1.0 alloy. X-ray diffraction (XRD) and scanning electron microscopy (SEM) show that the increase in Ni content is beneficial for promoting the formation of the face-centered-cubic (FCC) phase, and the body-centered-cubic (BCC) phase is gradually divided by the FCC phase. Electron backscatter diffraction (EBSD) shows that, with the increase in Ni content, the number of grain boundaries in the alloy is greatly reduced and numerous phase boundaries appear in the alloys. The degree of strain concentration is significantly reduced with the increasing Ni content. The corrosion mechanism of alloys is also discussed in this paper. Our study provides a method to balance the soft magnetic properties and corrosion resistance, paving the way for potential applications of Fe-Co-Ni-Al MEAs in corrosive environments.

## 1. Introduction

High-entropy alloys (HEAs) are composed of five or more elements in near-equiatomic proportions [1,2,3] that have simple phase structures [4,5,6], such as face-centered cube (FCC), body-centered cube (BCC) and hexagonal close-packed (HCP). Over the past few decades, HEAs have attracted considerable attention due to their excellent properties, including high strength, outstanding hardness and good wear resistance [7,8,9,10,11,12,13]. Numerous HEAs have been reported that exhibit magnetic properties, such as magneto-thermal effects [14,15,16], electromagnetic wave-absorbing properties [17,18], hard magnetic properties [19,20,21] and soft magnetic properties (SMPs) [22,23,24,25,26]. In recent years, ternary and quaternary medium-entropy alloys (MEAs) have also exhibited excellent SMPs. Rao et al. [27] found that FeCoNi MEA has a higher saturation magnetization (*M_s_* = 160 Am^2^/kg). The FeCoNiAl_0.2_ MEA studied by Tan et al. [28] has higher maximum permeability (*μ_m_* = 1574) and lower coercivity (*H_c_* = 70 A/m).

Corrosion of soft magnetic materials during service can significantly impact their performance and service life. Specifically, soft magnetic devices, such as transformers, generators, and sensors, are susceptible to surface corrosion when constantly exposed to humid air or saline solutions, which results in material failures and increases costs. Therefore, it is important to improve the corrosion resistance of soft magnetic MEAs. The addition of corrosion-resistant elements is an effective means of improving corrosion resistance [29,30,31,32,33]. For instance, studies by Li et al. [34] demonstrated that the incorporation of Cr to a Fe_1.25_CoNi_1.25_Al_0.25_ alloy improves its corrosion resistance. Dai et al. [35] identified that Mo significantly increased breakdown potential, giving CoCrFeNiMo_0.2_ HEA good corrosion resistance. However, added elements such as Cr, Cu and Mo are non-ferromagnetic and may impair the SMPs of MEAs [36,37]. Li et al. [34] found that in Fe_1.25_CoNi_1.25_Cr*_x_*Al_0.25_ MEAs, as the Cr content increased from 0.5 to 0.75, the *M_s_* of the alloy decreased from 52 to 17 Am^2^/kg. Şimşek et al. [38] discovered that adding Mo to FeCoNi alloy to form FeCoNiMo alloy would reduce its *M_s_* from 102 to 72 Am^2^/kg.

Our previous work has found that FeCoNiAl MEA exhibits good SMPs and mechanical properties [28]. In this work, we chose to increase the content of the ferromagnetic element Ni on the basis of FeCoNiAl MEA and study the SMPs, corrosion resistance and microstructure of alloys in the hope of achieving a trade-off between corrosion resistance and SMPs. There are three reasons for increasing Ni: firstly, as a ferromagnetic element, Ni is known to improve the soft magnetic parameters of alloys [39]. However, adding too much Ni will reduce the *M_s_* of the alloy [40]; secondly, Ni is able to strengthen the corrosion resistance properties of alloys as a passivating and stabilizing element [41,42]; and thirdly, as the main element, the atomic radius of Ni is similar to that of other major elements, which helps to prevent lattice distortion [43].

## 2. Materials and Methods

In this study, the nominal composition of the alloys is FeCoNi*_x_*Al (*x* = 1.0, 1.25, 1.5, 1.75, 2). The alloys were prepared by melting high-purity metals (purity > 99.9 wt.%) in a WK-II vacuum arc-melting furnace under an argon atmosphere. The melting current used in the melting process is 80 A and the chamber pressure is 0.115 MPa. Subsequently, an ingot with a mass of 15 g was melted four times and sucked into a water-cooled copper mold to obtain a 100 mm × 10 mm × 2 mm sheet sample. Electrochemical impedance spectra (EIS) and polarization techniques (Tafel) were performed in an electrochemical workstation (CHI900D; Chinstruments, Shanghai, China) at room temperature with 3.5 wt.% NaCl solution; the sample size for the electrochemical test was 10 mm × 10 mm, and it was prepared by wire cutting. The dynamic polarization curve was measured at a scan rate of 1 mV/s with an applied potential voltage of −0.5 to +0.5 V. EIS measurements were made in the potential mode of the open-circuit potential (OCP) with a frequency range of 100 kHz to 0.01 Hz. In this experiment, the reference and counter electrode were a saturated calomel electrode (SCE) and platinum sheet, respectively. *M_s_* was tested by vibrating a sample magnetometer (VSM; Lakeshore 7407; Westerville, OH, USA) under an applied magnetic field of 1.8 T. Alternating current (AC) SMPs and direct current (DC) SMPs of the specimens were measured using a soft magnetic measuring device (FE-2100SA; FE-2100SD; Forever elegance, Loudi, China). The resistivity of the alloys was determined by ST-2358C multifunctional four-probe resistivity (Jingge Electronic, Suzhou, China). Vickers hardness was tested with a Vickers hardness tester (HXD-1000TMC/LCD; Sunny Hengping Scientific Instrument, Shanghai, China) at 0.98 N for 10 s, and each sample was tested in nine different areas. To ensure data reliability, the maximum and minimum values were removed, and the average of the remaining values was then taken as the Vickers hardness of the alloy. The crystal structure was obtained by X-ray diffraction (XRD; D/max 2500 V; Rigaku Corporation, Akishima-Shi, Japan) with Cu Kα radiation; the sample size for the XRD test was 24 mm × 9.6 mm × 1.8 mm, which was scanned from 20° to 100° at a scanning rate of 1°/min; the detector used was a PIXCEL 1D with a step size set to 0.02°. The microstructure of the alloys was observed by Tescan Mira 3 and Hitachi SU8700 scanning electron microscopy (SEM; Bruker e-FlashFS, Bruker Nano GmbH, Hamburg, Germany) with energy dispersive spectrometry (EDS) and electron backscatter diffraction (EBSD). The accelerating voltage is set to 15 kV and 20 kV, respectively. In addition, the FCC phase space group used in the EBSD experiment is Fm–3m (225), while the BCC phase space group is Im–3m (229).

## 3. Results

### 3.1. Corrosion Properties

**Figure 1:**
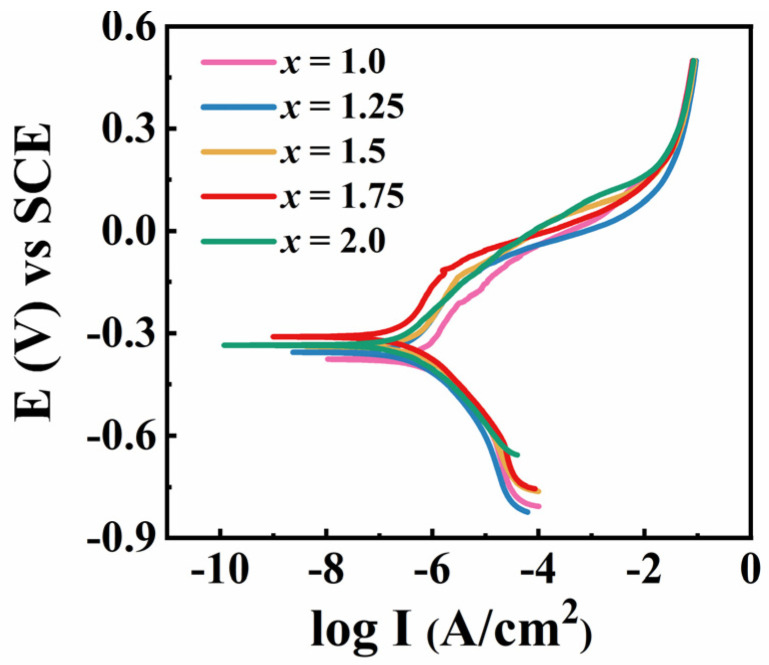
The potential dynamic polarization curves of FeCoNi*_x_*Al (*x* = 1.0–2.0) MEAs in 3.5 wt.% NaCl solution.

Figure 1 shows the potential dynamic polarization curves of FeCoNi*_x_*Al (*x* = 1.0–2.0) MEAs in 3.5 wt.% NaCl solution. It is observed that the polarization curves of all alloys show a very narrow passivation zone in the range of −0.4 to 0 V, indicating that the passivation film was formed. The anodic polarization curve shifts significantly to the left with an increase of Ni, implying that the polar dissolution rate slowed down. However, the cathodic polarization curve basically overlapped, indicating that the cathodic reaction rate is not affected by the Ni content.

The values of corrosion potential (*E_corr_*) and corrosion current density (*I_corr_*) of FeCoNi*_x_*Al (*x* = 1.0–2.0) MEAs in 3.5 wt.% NaCl solution are listed in Table 1. For comparison, the *E_corr_* and *I_corr_* of some reported MEAs, amorphous alloys and 304 stainless steel under the same conditions are also given. The *I_corr_* of the *x* = 1.0 alloy is 9.12 × 10^−7^ A/cm^2^, which is better than for 304 stainless steel and some MEAs containing Cr. The *I_corr_* of the alloys reduces monotonically with the increase in Ni content. When *x* = 1.25, the *I_corr_* is 4.28 × 10^−7^ A/cm^2^, which is significantly reduced by 53% compared to the *x* = 1.0 alloy. The *x* = 2.0 alloy has the lowest *I_corr_* (2.67 × 10^−7^ A/cm^2^), which is a 71% improvement compared to the *x* = 1.0 alloy. The *E_corr_* of the alloys rises initially and then falls slightly as Ni increases. When *x* = 1.75, the *E_corr_* value of the alloy is the largest (−0.31 V), which is lower than pure Ni and 304 stainless steel, but better than pure Ti and some MEAs. The above results indicate that an appropriate increase of Ni content is effective in enhancing the corrosion resistance of FeCoNi*_x_*Al MEAs.

**Figure 2:**
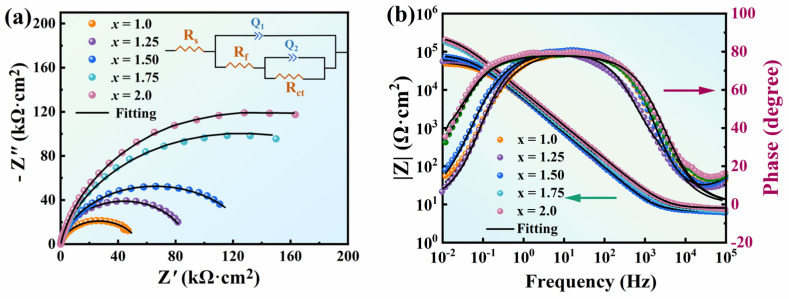
EIS plots of FeCoNixAl (*x* = 1.0–2.0) MEAs in 3.5 wt.% NaCl solution. (**a**) Nyquist plots; (**b**) Bode plots; (Inset in (**a**) is the EEC).

Figure 2 shows the EIS of FeCoNi*_x_*Al (*x* = 1.0–2.0) MEAs measured at open circuit potential. As shown in Figure 2a, the Nyquist curves of the alloys are semicircular. The capacitance arc radius increases with the increase in Ni content, indicating that corrosion resistance is enhanced [51]. Figure 2b displays the Bode plots, where the slopes of all samples are close to −1 in the frequency range of 10^0^–10^3^ Hz, suggesting that the passivation film has an electrochemical reaction [52]. However, the slopes of the curves change in the range of 10^−2^–10^0^, indicating that the electrochemical behavior of the double layer has changed [53]. In addition, the phase angle of *x* = 1.0–2.0 alloys is less than 90° in the mid-frequency region, implying non-ideal pure capacitive behavior [54]. It is observed that the phase angle for 1.0 ≤ *x* ≤ 1.5 alloys is always lower than those for alloys with *x* = 1.75 and *x* = 2.0 in the range of 10^−2^–10^0^ Hz, indicating that the dissolution rate of the passivation film for the *x* = 1.75 and 2.0 alloys is lower [55].

Based on the Nyquist plots, the electrode process is controlled by the charge transfer process. The variation in impedance slope at low frequencies indicates that there are two time constants in these MEAs [56]. The corresponding equivalent electric circuit (EEC) model is shown in the inset of Figure 2a, where *R_s_* represents the resistance of the electrolyte solution and *Q*_1_ is the passive film capacitance, which is coupled to the passive film resistance *R_f_*. Moreover, *Q*_2_ and *R_ct_* correspond to the electrical double-layer capacitance and the charge transfer resistance, respectively. Table 2 details the fitting parameters. The variance between the fitting data and the experimental data was within 10^−3^, which indicates a good fit. The values of *Q*_1_ and *Q*_2_ decrease, implying that the corrosion resistance of alloys is improved [57]; *R_p_* is the sum of *R_f_* and *R_ct_*, and the higher the value of *R_p_*, the better the corrosion resistance of the alloy. For the system of FeCoNi*_x_*Al (*x* = 1.0–2.0), *R_p_* increases as *x* increases, which is consistent with the variation in *I_corr_* for the alloys.

### 3.2. X-Ray Diffraction

**Figure 3:**
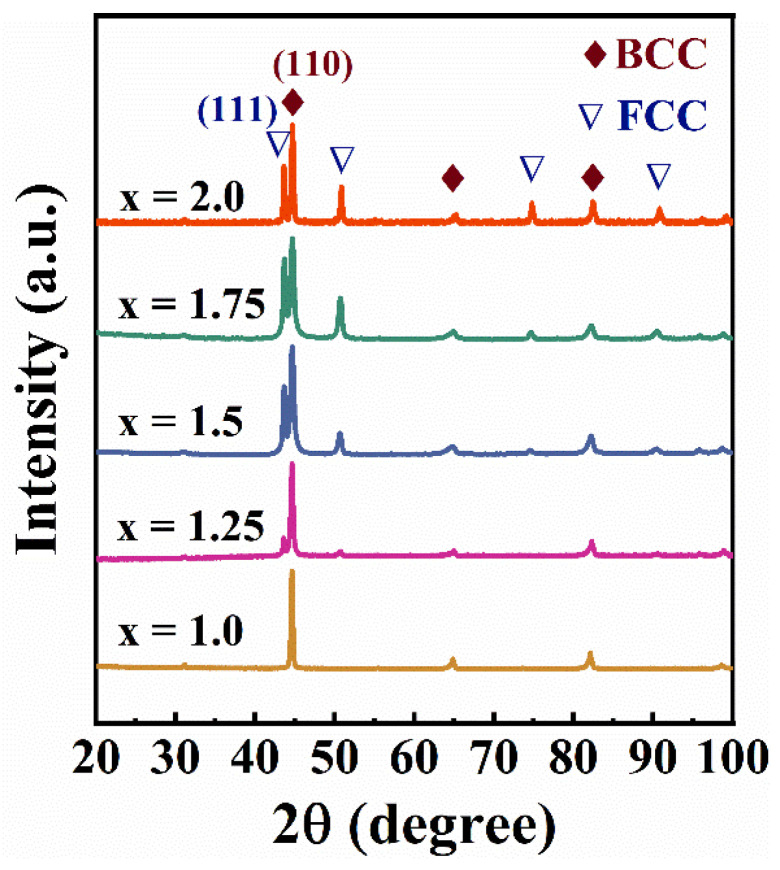
The XRD patterns of FeCoNi*_x_*Al (*x* = 1.0–2.0) MEAs.

Figure 3 shows the XRD patterns of FeCoNi*_x_*Al (*x* = 1.0–2.0) MEAs. It is found that there is only a BCC phase in the *x* = 1.0 alloys, whereas the *x* = 1.25 alloys consist of FCC phase and BCC phases. As the Ni content increases, the relative intensity of the FCC phase diffraction peaks increases.

### 3.3. Magnetic Properties

**Figure 4:**
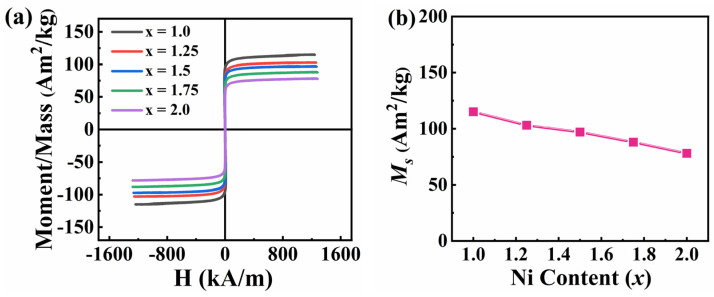
Magnetic properties of FeCoNi*_x_*Al (*x* = 1.0–2.0) MEAs under an applied magnetic field of 1.8 T. (**a**) hysteresis loops; (**b**) curves of *M_s_* as a function of Ni content.

Figure 4a illustrates the hysteresis loops of FeCoNi*_x_*Al (*x* = 1.0–2.0) MEAs, and all alloys have soft magnetic behavior. Figure 4b shows the curves of *M_s_* with the Ni content. It is found that the *M_s_* of the alloy decreases linearly as *x* increases, and there could be two reasons for this: the *M_s_* of Ni (54.38 Am^2^/kg) is smaller than that of the *x* = 0 alloy (115 Am^2^/kg); and the phase composition of the alloy changes with the increase in Ni content. The *M_s_* of the FCC phase is reported to be different from that of the BCC phase [58]. In addition, the DC soft magnetic parameters of the alloy are shown in Table S1, from which it is found that the initial permeability (*μ_i_*) and *μ_m_* of the alloy decrease with the increase in Ni content.

**Figure 5:**
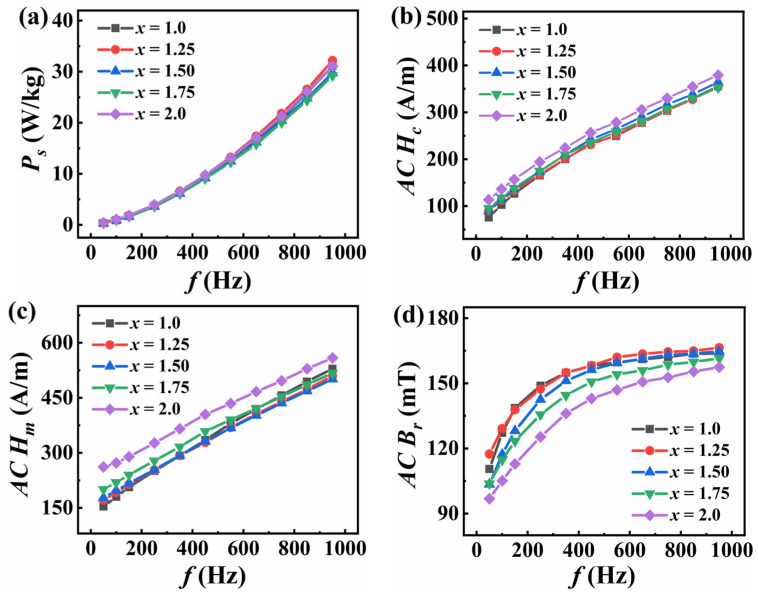
Curves of AC soft magnetic parameters for FeCoNi*_x_*Al (*x* = 1.0–2.0) MEAs with frequency at *B_m_* = 200 mT. (**a**) *P_s_*; (**b**) *AC B_r_*; (**c**) *AC H_m_*; (**d**) *AC H_c_*.

Figure 5 shows the curves of total loss (*P_s_*), AC remanence (*AC B_r_*), maximum magnetic field in AC (*AC H_m_*) and AC coercivity (*AC H_c_*) of FeCoNi*_x_*Al (*x* = 1.0–2.0) MEAs with frequency (*f*) at *B_m_* = 200 mT. All AC soft magnetic parameters are positively correlated with *f*.

**Figure 6:**
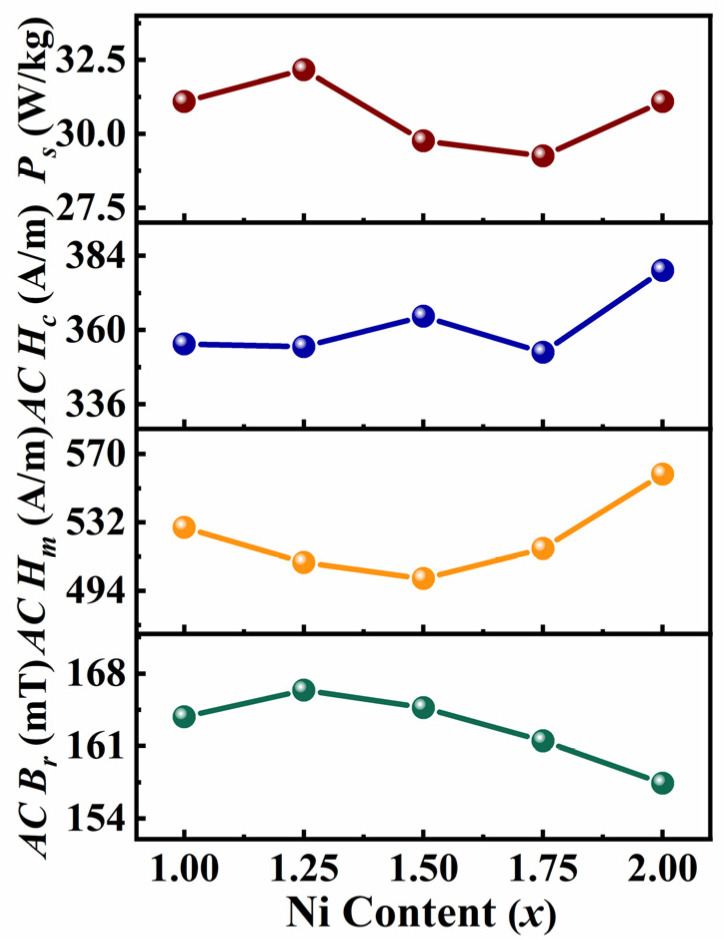
Curves of AC soft magnetic parameters for FeCoNi*_x_*Al (*x* = 1.0–2.0) MEAs as a function of Ni content (*f* = 950 Hz).

In order to further explore the changes of each magnetic parameter, the variation curves of AC parameters with Ni content at *f* = 950 Hz are plotted, as shown in Figure 6, and Table 3 gives the relevant values. The *AC B_r_* increases initially and then decreases with the increase in Ni content, and the minimum value of 157 mT is obtained at *x* = 2.0. The *AC H_m_* first decreases and then increases with the increasing Ni content, and the *AC H_m_* of the *x* = 1.5 alloy is the lowest (501 A/m). The *AC H_c_* and *P_s_* fluctuate as *x* increases, and the *x* = 1.75 alloy has the minimum values of *AC H_c_* and *P_s_*, which are 353 A/m and 29.25 W/kg, respectively. In summary, by increasing the Ni content, the AC SMPs of FeCoNi*_x_*Al HAEs can be slightly improved, with a 6% improvement in *P_s_* in the *x* = 1.75 alloy compared to the *x* = 1.0 alloy. Compared with some other alloy systems (see Table 3), FeCoNi*_x_*Al (*x* = 1.0–2.0) MEAs has good SMPs.

### 3.4. Resistivity and Hardness

**Figure 7:**
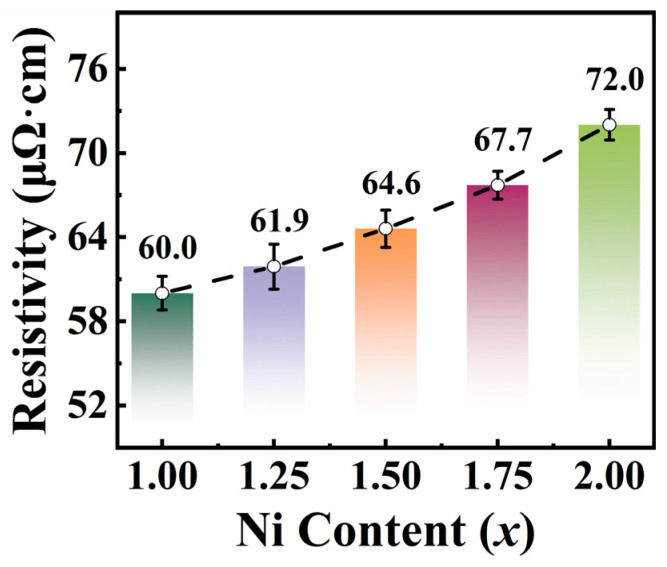
The resistivity for FeCoNi*_x_*Al (*x* = 1.0–2.0) MEAs as a function of Ni content.

Figure 7 shows the resistivity (*ρ*) for FeCoNi*_x_*Al (*x* = 1.0–2.0) MEAs as a function of Ni content. The relevant values are listed in Table 4. As the Ni content increases, resistivity increases linearly. Based on the XRD results, the 1.25 ≤ *x* ≤ 2.0 alloys probably have phase boundaries that prevent the movement of electrons, which could result in electron scattering and then increase the resistivity of the alloys [62]. Generally, resistivity is inversely proportional to eddy current loss (*P_e_*) [63], and the *P_s_* mainly includes hysteresis loss (*P_h_*) and *P_e_*. Thus, an increase in resistivity is beneficial to reduce *P_s_* of alloys.

**Figure 8:**
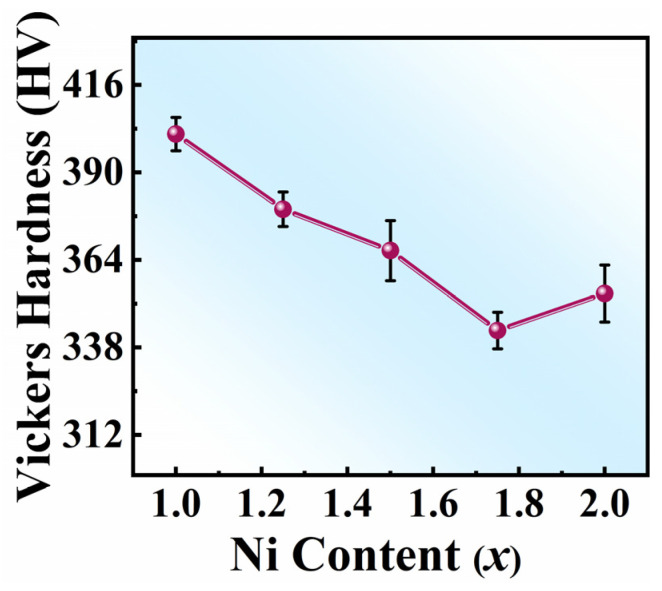
The Vickers hardness for FeCoNi*_x_*Al (*x* = 1.0–2.0) MEAs as a function of Ni content.

Figure 8 shows the Vickers hardness for FeCoNi*_x_*Al (*x* = 1.0–2.0) MEAs as a function of Ni content. The relevant values are listed in Table 4. As the Ni content increases, the Vickers hardness decreases first and then increases. In MEAs and HEAs, the FCC phase exhibits greater ductility but lacks strength, whereas the BCC phase has higher strength but less ductility [64,65]. As the Ni content increases, the Vickers hardness of the alloy undergoes an initial decrease followed by a slight increase, which is closely related to the volume fraction variations of the FCC and BCC phases in the alloy.

### 3.5. Microstructure

**Figure 9:**
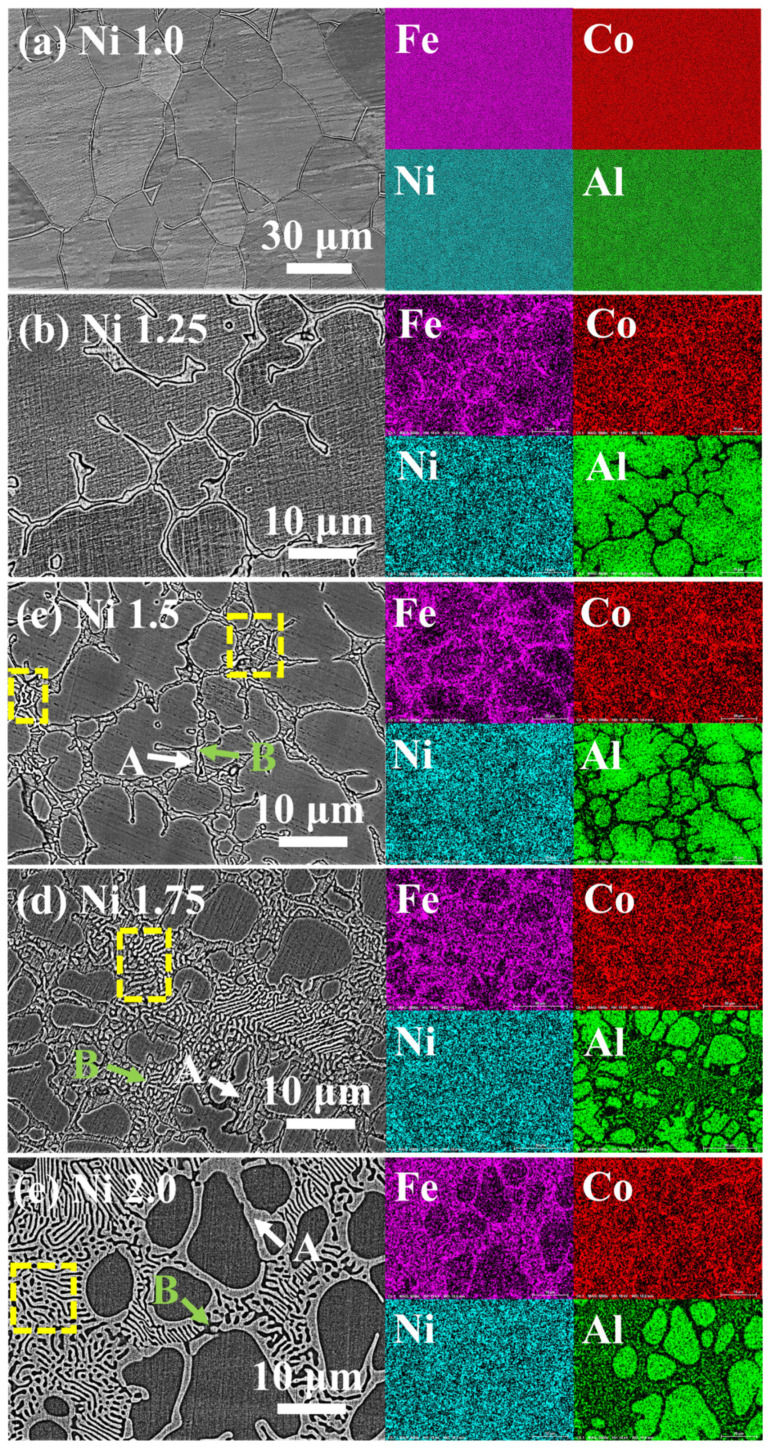
SEM-BSE images and SEM-EDS maps of FeCoNi*_x_*Al (*x* = 1.0–2.0) MEAs. (**a**) *x* = 1.0; (**b**) *x* = 1.25; (**c**) *x* = 1.5; (**d**) *x* = 1.75; (**e**) *x* = 2.0.

Figure 9 displays the SEM backscattered electron (BSE) images and EDS maps of FeCoNi*_x_*Al (*x* = 1.0–2.0) MEAs. From Figure 9a, it is observed that the *x* = 1.0 alloy is composed of equiaxed crystals. According to the XRD results, the *x* = 1.0 alloy has the structure of the BCC phase. The EDS results show all elements uniformly distributed. When *x* = 1.25, a new network structure appears in the dark gray matrix, as shown in Figure 9b. Based on the XRD and EDS results, it can be determined that the dark gray matrix rich in Al is the BCC phase, whereas the light gray network rich in Fe and Co is the FCC phase. When *x* = 1.5, a dark gray matrix of the BCC phase and an alternating area of two different contrasts can be observed, as shown in Figure 9c. The regions in the eutectic lamellar structure (marked by the yellow-dashed box) near the dark gray matrix of the BCC phase are marked A (marked with a white arrow), and the other one is labeled B (marked with a green arrow). From the map results, Al is enriched in the BCC phase and Region B, whereas Fe and Co are enriched in Region A. From Figure 9d,e, it can be seen that as the Ni content increases, the area of the eutectic lamellar structure gradually increases. The EDS maps suggest that Al is enriched in the BCC phase and Region B, whereas Fe and Co are enriched in Region A, similar to the *x* = 1.50 alloy.

The point-scanning results of FeCoNi*_x_*Al (*x* = 1.0–2.0) MEAs are given in Table 5, which are consistent with the mappings in Figure 9. When 1.5 ≤ *x* ≤ 2.0, Region A is enriched in Fe and Co but poor in Al, which is similar to the FCC phase in the *x* = 1.25 alloy. Furthermore, the elemental distribution of Region B is similar to the nominal composition.

**Figure 10:**
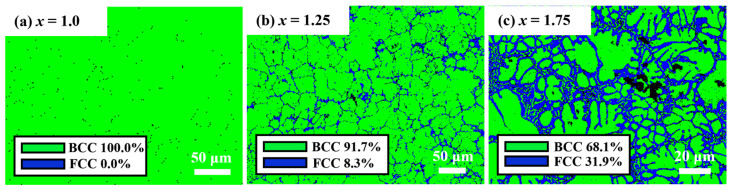
Phase maps of the FeCoNi*_x_*Al MEAs. (**a**) *x* = 1.0; (**b**) *x* = 1.25; (**c**) *x* = 1.75.

In order to further analyze the phase composition of the alloy, *x* = 1.0, 1.25 and 1.75 samples are selected for EBSD, as shown in Figure 10. When *x* = 1.0, the alloy consists of a BCC phase and an FCC phase with a volume fraction of nearly 0%. Due to the very small amount of FCC phase, it cannot be observed in XRD and SEM results. When *x* = 1.25, an FCC phase network structure is observed within the BCC phase, with a volumetric fraction of 8.3%. When *x* = 1.75, there is a notable expansion in the area of the FCC phase, reaching a volume fraction of 31.9%, and the BCC structure is segmented more finely. In addition, strips of BCC phase emerge within the FCC phase, confirming that Region A near the BCC matrix is the FCC phase and that Region B is the BCC phase (called BCC′), respectively. In other words, the eutectic lamellar structure is composed of the FCC phase and the BCC′ phase. Additionally, there is no obvious anisotropy in the grain orientation (as shown in Figure S1).

**Figure 11:**
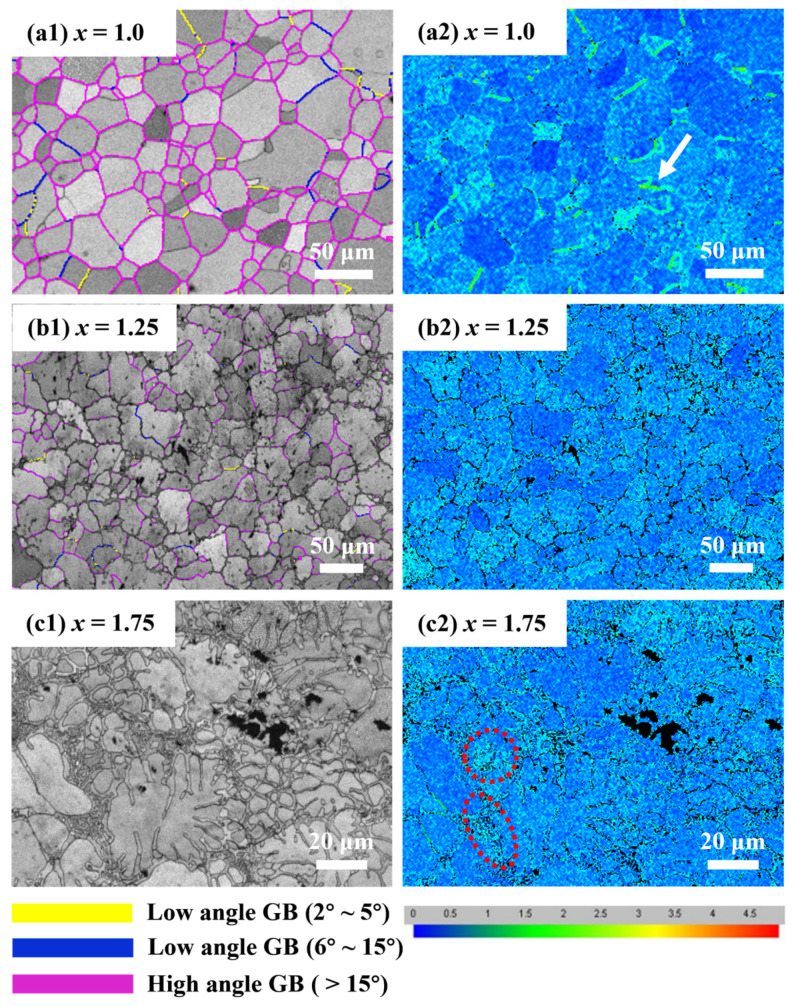
GBCD maps and KAM maps of the FeCoNi*_x_*Al MEAs. (**a1**,**a2**) *x* = 1.0; (**b1**,**b2**) *x* = 1.25; (**c1**,**c2**) *x* = 1.75.

Figure 11 illustrates the grain boundary characteristic distribution (GBCD) maps and kernel average misorientation (KAM) maps of FeCoNi*_x_*Al (*x* = 1.0, 1.25, 1.75) MEAs. When *x* = 1.0, there are numerous high angle grain boundaries (GBs, >15°) while low angle GBs (<15°) are relatively few. The strain is mainly concentrated at the low angle GBs of 2°~5° (marked as the white arrow in Figure 11(a2)). When *x* = 1.25, the number of high angle GBs decrease, and no significant strain concentration is found at the GBs. When *x* = 1.75, almost no GBs are observed in the alloy, and a large number of phase boundaries between the FCC and BCC phases appear. The average orientation difference between the alloy grains decreases and the strain is only concentrated within the layer-like region (marked as the red circle in Figure 11(c2)).

## 4. Discussion

**Figure 12:**
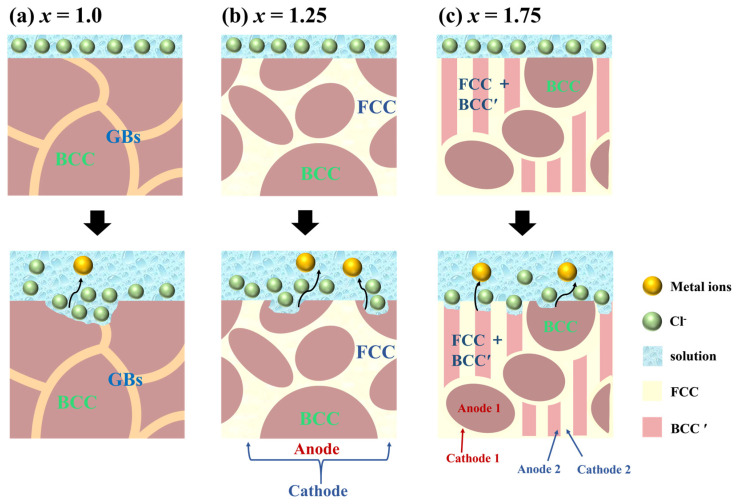
Corrosion mechanism of FeCoNi*_x_*Al MEAs in 3.5 wt.% NaCl solution; (**a**) *x* = 1.0, (**b**) *x* = 1.25, (**c**) *x* = 1.75.

From the above results, it can be found that increasing the Ni content appropriately in FeCoNiAl MEAs can significantly improve corrosion resistance without reducing the AC SMPs. We have proposed a schematic representation elucidating the corrosion mechanism of the alloy system based on its performance and microstructure, as shown in Figure 12. When *x* = 1.0, the alloy consists of BCC phase with a volume fraction of nearly 100%, as depicted in Figure 12a. Uniform corrosion occurs due to the uniform homogeneity of elements in the alloy. However, because there are numerous GBs and the strain is concentrated at the lowangle GBs, the *x* = 1.0 alloy has a fast corrosion rate.

For the *x* = 1.25 alloy, *I_corr_* is 4.28 × 10^−7^, which is a 53% reduction compared to the *x* = 1.0 alloy. The reasons for improvement in corrosion resistance are as follows: the *x* = 1.25 alloy is composed of BCC and FCC phases, but the FCC structure exhibits superior resistant than the BCC structure, as seen by austenitic stainless steels which demonstrates enhanced corrosion resistance in comparison to ferritic stainless steels [66,67]; and no significant strain concentration is found at GBs for the *x* = 1.25 alloy, which results in a decrease in electrochemical activity of the alloy, thereby improving the corrosion resistance [68]. Finally, there is micro-galvanic corrosion formed between the BCC and FCC phases. The BCC phase contains a higher concentration of Al, while Cl^−^ easily forms metastable ion complexes with Al to increase the electrochemical activity of the alloy [69]. Thus, the structure of BCC is dissolved at the anode in an oxidation reaction with Cl^−^, as illustrated in Figure 12b.

When *x* = 1.75, *I_corr_* further reduces to 3.08 × 10^−7^ A/cm^2^, which is mainly attributed to the elevated volume fraction of the FCC phase [66,67]. Since the strain is mainly concentrated in the eutectic lamellar structure, the eutectic lamellar structure is susceptible to corrosion, resulting in localized corrosion [68]. In addition, the *x* = 1.75 alloy exhibits two types of micro-galvanic corrosion. One is the galvanic coupling between the BCC and FCC phases, and its corrosion process is similar to the *x* = 1.25 alloy. The other is in the eutectic lamellar structure, as illustrated in Figure 12c. Due to the alternating distribution of the BCC′ and FCC phases, numerous micro-galvanic cells are formed in this area. However, the differences in the content of Fe, Co, Ni and Al between the BCC/FCC and BCC′/FCC phases are significantly reduced. This compositional similarity between the phases weakens the driving force for galvanic interactions, resulting in a decreased rate of galvanic corrosion [70,71].

## 5. Conclusions

In summary, the corrosion resistance, AC SMPs and microstructure of FeCoNi*_x_*Al (*x* = 1.0–2.0) MEAs were investigated, and the main results are as follows. Corrosion resistance is greatly improved with increasing Ni content, and the *x* = 2.0 alloy has a corrosion current density (*I_corr_*) of 2.67 × 10^−7^ A/cm^2^, which is reduced by 71% compared to the *x* = 1.0 alloy. Increasing the content of Ni can improve the AC SMPs of the alloy. When *x* = 1.75, the total loss (*P_s_*) is improved by 6% compared to the *x* = 1.0 alloy. The Ni element is beneficial to promote the formation of the FCC phase, and the BCC phase is gradually divided and refined by the FCC phase with increasing Ni content. The EBSD results show that the *x* = 1.0 alloy has numerous high-angle GBs (>15°) but few low-angle GBs (<15°). The strain is mainly concentrated at the low-angle GBs of 2–5°. And increasing the Ni content reduces the number of GBs and decreases the strain concentration of the alloy. Our findings provide a way to balance the SMPs and corrosion resistance and help to clarify the corrosion mechanism of soft magnetic MEAs.

## Figures and Tables

**Table 1:** Electrochemical parameters of FeCoNi*_x_*Al (*x* = 1.0–2.0) MEAs, pure metals and typical alloys in 3.5 wt.% NaCl solution.

Alloys	*I_corr_* (A/cm^2^)	*E_corr_* (V)	Ref.
FeCoNiAl	9.12 × 10^−7^	−0.38	Current
FeCoNi_1.25_Al	4.28 × 10^−7^	−0.36	Current
FeCoNi_1.50_Al	4.16 × 10^−7^	−0.34	Current
FeCoNi_1.75_Al	3.08 × 10^−7^	−0.31	Current
FeCoNi_2_Al	2.67 × 10^−7^	−0.34	Current
Ti	2.51 × 10^−7^	−0.37	[44]
Ni	1.20 × 10^−6^	−0.18	[45]
FeSiB amorphous alloy	2.18 × 10^−6^	−0.77	[46]
304 stainless steels	(1.99)–(3.16) × 10^−6^	(−0.22)–(0.27)	[47]
FeCoNi_2.1_CrAl	7.31 × 10^−7^	−0.27	[48]
FeCoNiCr*_x_* (*x* = 0–1.0)	(2.36)–(7.05) × 10^−6^	(−0.41)–(−0.32)	[29]
FeCoNiCrAl*_x_* (*x* = 0.3–0.7)	(0.84)–(4.29) × 10^−7^	(−0.28)–(−0.20)	[31]
FeCoNiCuAlCe*_x_* (*x* = 0–0.09)	(4.01)–(5.78) × 10^−6^	(−0.45)–(−0.43)	[49]
FeCoNiAlCrTi*_x_* (*x* = 0.5–2.0)	(0.73)–(4.70) × 10^−7^	(−0.18)–(−0.23)	[50]

**Table 2:** Equivalent circuit fitting data for FeCoNi_x_Al (*x* = 1.0~2.0) MEAs.

Alloys	*R_s_* (ohm)	*Q*_1_ (10^−5^)	*R_f_* (kohm)	*Q*_2_ (10−4)	*R_ct_* (kohm)	*R_p_* (kohm)
*x* = 1.0	7.94	2.80	0.02	0.27	55.21	55.23
*x* = 1.25	7.90	2.13	37.5	0.26	60.76	98.26
*x* = 1.50	7.51	2.11	71.3	0.27	85.85	157.15
*x* = 1.75	7.26	1.89	84.3	0.29	175.66	259.96
*x* = 2.0	7.96	1.56	105.0	0.20	228.23	333.23

**Table 3:** *M_s_* (*H_m_* = 1.8 T), AC soft magnetic parameters (*f* = 950 Hz) of FeCoNi*_x_*Al (*x* = 1.0–2.0) MEAs and some alloys reported in the references.

Alloys	*M_s_* (Am^2^/kg)	*P_s_* (W/kg)	*AC H_c_* (A/m)	*AC H_m_* (A/m)	*AC B_r_* (mT)	Ref.
FeCoNiAl	115	31.09	355	529	164	Current
FeCoNi_1.25_Al	103	32.17	355	510	166	Current
FeCoNi_1.50_Al	97	29.76	364	501	165	Current
FeCoNi_1.75_Al	88	29.25	353	517	162	Current
FeCoNi_2_Al	78	31.09	379	559	157	Current
FeCoNi(MnSi)_x_ (*x* = 0–0.4)	59–138	16.93–26.84	272–462	-	-	[25]
FeCoNi_1+*x*_Cu_1−*x*_Al (*x* = 0–1.0)	79–82	37.65–85.32	433–1044	617–1664	130–168	[26]
FeCoNiCuMn	84	-	-	-	-	[59]
FeCoNiAlCr	70	-	-	-	-	[60]
FeNi	84	-	-	-	-	[61]

**Table 4:** Resistivity and Vickers hardness of FeCoNi_x_Al (*x* = 1.0~2.0) MEAs.

Alloys	Resistivity (μΩ·cm)	Vickers Hardness (HV)
FeCoNiAl	60.0 ± 1.2	401 ± 5
FeCoNi_1.25_Al	61.9 ± 1.6	379 ± 5
FeCoNi_1.50_Al	64.6 ± 1.3	367 ± 9
FeCoNi_1.75_Al	67.7 ± 1.0	343 ± 5
FeCoNi_2_Al	72.0 ± 1.1	354 ± 8

**Table 5:** Point-scanning results of FeCoNi*_x_*Al (*x* = 1.0~2.0) MEAs (at%).

*x*	Regions	Fe	Co	Ni	Al
1.0	Nominal	25	25	25	25
	BCC	25.26 ± 0.56	26.35 ± 1.05	24.74 ± 3.09	23.26 ± 2.85
1.25	Nominal	23.53	23.53	29.41	23.53
	BCC	24.24 ± 0.37	24.82 ± 0.26	28.91 ± 0.54	22.04 ± 0.63
	FCC	32.17 ± 0.13	28.07 ± 0.30	27.24 ± 0.22	12.54 ± 0.39
1.5	Nominal	22.22	22.22	33.33	22.22
	BCC	18.54 ± 0.55	21.41 ± 0.23	34.36 ± 0.26	25.70 ± 0.20
	Region A	28.22 ± 0.68	26.23 ± 0.87	31.73 ± 0.42	13.84 ± 0.39
	Region B	23.19 ± 1.37	23.42 ± 0.59	33.11 ± 0.81	20.30 ± 1.25
1.75	Nominal	21.05	21.05	36.84	21.05
	BCC	19.25 ± 1.02	20.67 ± 1.67	37.59 ± 0.19	22.55 ± 0.28
	Region A	24.99 ± 0.63	24.33 ± 0.46	36.12 ± 0.35	14.56 ± 0.92
	Region B	20.51 ± 0.77	21.12 ± 0.89	38.16 ± 0.77	20.21 ± 1.33
2.0	Nominal	20	20	40	20
	BCC	17.04 ± 0.86	19.96 ± 0.79	41.26 ± 0.24	21.74 ± 1.07
	Region A	22.81 ± 0.32	22.93 ± 0.90	39.93 ± 0.56	14.33 ± 0.77
	Region B	19.45 ± 0.49	20.71 ± 0.24	40.73 ± 0.85	19.45 ± 0.69

## Data Availability

The data presented in this study are available on request from the corresponding author.

## Supplementary Materials

The following supporting information can be downloaded at: https://www.mdpi.com/article/10.3390/e26121038/s1, Figure S1: Inverse pole figure (IPF) of FeCoNi*_x_*Al MEAs. (a) *x* = 1.0; (b) *x* = 1.25; (c) *x* = 1.75. Table S1. DC soft magnetic parameters of FeCoNi*_x_*Al (*x* = 1.0–2.0) MEAs.
