# Supplementary material for: Tuning Corrosion Resistance and AC Soft Magnetic Properties of Fe-Co-Ni-Al Medium-Entropy Alloy via Ni Content
Source: Entropy (Basel). 2024 Nov 30;26(12):1038. doi: 10.3390/e26121038 (PMC11727253; doi:10.3390/e26121038)
Supplement: Supplementary file 1 [file entropy-26-01038-s001.zip › entropy-3244241-supplementary.pdf]

**Table S1.** DC soft magnetic parameters of FeCoNi$_x$Al ($x$ = 1.0–2.0) MEAs.

| Alloys | $\mu_i$ | $\mu_m$ | $B_r$ (T) | $H_c$ (A/m) | $P_u$ (J/m$^3$) |
|---|---|---|---|---|---|
| FeCoNiAl | 363 | 1249 | 0.230 | 77 | 244 |
| FeCoNi$_{1.25}$Al | 320 | 1059 | 0.239 | 91 | 307 |
| FeCoNi$_{1.50}$Al | 382 | 974 | 0.267 | 116 | 399 |
| FeCoNi$_{1.75}$Al | 334 | 828 | 0.264 | 135 | 453 |
| FeCoNi$_2$Al | 310 | 647 | 0.282 | 193 | 509 |

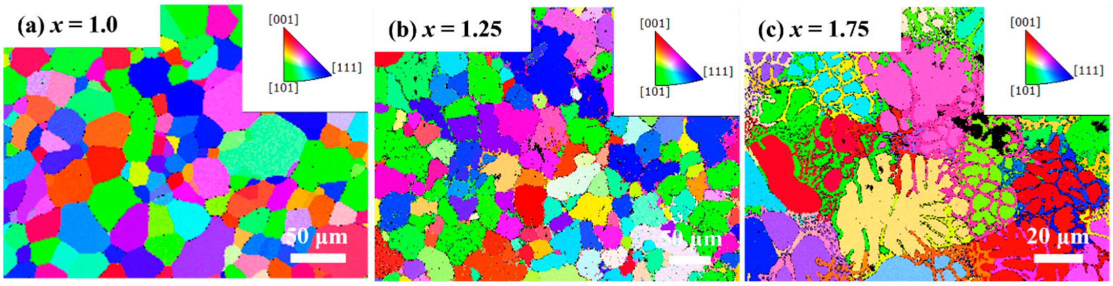


**Figure S1.** Inverse pole figure (IPF) of FeCoNi$_x$Al MEAs. (a) $x$ = 1.0; (b) $x$ =1.25; (c) $x$ = 1.75.
